# Conserved Threonine Residues within the A-Loop of the Receptor NIK Differentially Regulate the Kinase Function Required for Antiviral Signaling

**DOI:** 10.1371/journal.pone.0005781

**Published:** 2009-06-03

**Authors:** Anésia A. Santos, Claudine M. Carvalho, Lilian H. Florentino, Humberto J. O. Ramos, Elizabeth P. B. Fontes

**Affiliations:** Departamento de Bioquímica e Biologia Molecular/BIOAGRO, Universidade Federal de Viçosa, Viçosa, Minas Gerais, Brazil; CNRS UMR 8079/Université Paris-Sud, France

## Abstract

NSP-interacting kinase (NIK1) is a receptor-like kinase identified as a virulence target of the begomovirus nuclear shuttle protein (NSP). We found that NIK1 undergoes a stepwise pattern of phosphorylation within its activation-loop domain (A-loop) with distinct roles for different threonine residues. Mutations at Thr-474 or Thr-468 impaired autophosphorylation and were defective for kinase activation. In contrast, a mutation at Thr-469 did not impact autophosphorylation and increased substrate phosphorylation, suggesting an inhibitory role for Thr-469 in kinase function. To dissect the functional significance of these results, we used NSP-expressing virus infection as a mechanism to interfere with wild type and mutant NIK1 action in plants. The NIK1 knockout mutant shows enhanced susceptibility to virus infections, a phenotype that could be complemented with ectopic expression of a 35S-NIK1 or 35S-T469A NIK1 transgenes. However, ectopic expression of an inactive kinase or the 35S-T474A NIK1 mutant did not reverse the enhanced susceptibility phenotype of knockout lines, demonstrating that Thr-474 autophosphorylation was needed to transduce a defense response to geminiviruses. Furthermore, mutations at Thr-474 and Thr-469 residues antagonistically affected NIK-mediated nuclear relocation of the downstream effector rpL10. These results establish that NIK1 functions as an authentic defense receptor as it requires activation to elicit a defense response. Our data also suggest a model whereby phosphorylation-dependent activation of a plant receptor-like kinase enables the A-loop to control differentially auto- and substrate phosphorylation.

## Introduction

The perception of external stimuli through cell surface receptors is a common mechanism of multicellular organisms that allows communication among cells and between cells and the external environment. In plants, an extensive battery of Ser/Thr receptor-like kinases (RLK) may transduce external signals into cells through the reversible phosphorylations that allow the cells to sense and respond to external signals in a precise, regulated and adaptive way [1].

In the Arabidopsis genome, the RLK family is represented by 417 sequences that are organized into a typical receptor configuration harboring an N-terminal extracellular domain followed by a transmembrane segment and a kinase domain at the C-terminus [1]. These receptors have been shown to be predominantly involved in developmental events and defense strategies. Characterized members of this family include BRI1 and BAK1 involved in brassinosteroid signaling [2], [3], [4], HAESA associated with floral abscission regulation [5], SERK1 associated with early embryogenesis [6], BONZAI responsible for growth capacity under different temperature conditions [7], ERECTA and CLAVATA1 that control size and shape of flowers [8], [9], RLK2 that controls anther development [10] and typical defense proteins, such as FLS2 [11], BAK1 [12], [13] and the rice Xa-21 protein [14].

The representatives of the Arabidopsis RLK superfamily have been phylogenetically organized into families based on the structural identity of extracellular domains and conservation of C-terminal kinase domains [1], [15], [16]. The major family comprises the leucine-rich repeats (LRR)-RLKs which contain three to 26 LRR motifs in their extracellular domains [1]. Further subdivision into 13 sub-families (LRRI-LRRXIII) is based on sequence identity and organization of the LRR domain. One of these, the LRRII-RLK subfamily, is constituted by 14 proteins harboring four complete LRRs (with 24 residues) and a fifth incomplete LRR (with 16 residues) arranged in a single continuous block within the extracellular domain [17]. Phylogenetic analysis based on sequence conservation and evolutionary structural features of LRRII-RLKs has demonstrated that the members of this subfamily are clustered into three distinct branches of functional relatedness: (i) antiviral defense proteins, (ii) developmental proteins and (iii) functionally unassigned proteins. The NSP-interacting kinase 1, NIK1 (At5g16000), NIK2 (At3g25560) and NIK3 (At1g60800) are inserted into the defense group I of the LRRII-RLK sub-family [17] and they have been initially identified as virulence targets of the bipartite geminivirus nuclear shuttle protein, NSP [18]. The NSP-NIK interaction is conserved among geminivirus NSPs and NIK homologs from different hosts. Tomato and soybean NIK homologs also interact stably with NSP from CaLCuV (*Cabbage leaf curl virus*) and from the tomato-infecting geminiviruses TGMV (*Tomato golden mosaic virus*) and TCrLYV [Tomato crinkle leaf yellows virus; 18], [19], [20]. The assignment of NIK as a defense receptor gene was based primarily on the enhanced susceptibility phenotype to geminivirus infection displayed by nik knockout lines [18]. Recent progress towards elucidating the NIK-mediated antiviral signaling includes the identification of the ribosomal protein L10 (rpL10) as the immediate downstream effector in the pathway [21], [22]. Phosphorylation of rpL10 by NIK promotes translocation of the ribosomal protein to the nucleus where it may function to mount a defense response that negatively impacts virus infection. The bipartite geminivirus NSP suppresses NIK activity through specific binding to the kinase domain and hence enhances geminivirus pathogenicity. Thus, upon geminivirus infection, rpL10 is trapped within the cytoplasm to prevent the establishment of a host environment that disfavors virus proliferation and/or spread.

Geminiviruses are considered one of the largest and most successful groups of plant viruses that infect a wide range of crops, particularly in tropical and subtropical regions. These plant viruses are characterized by single-stranded circular DNA genomes that are encapsidated in geminate semi-icosahedral particles and replicate via a double-stranded DNA intermediate in the nuclei of infected cells [for review see 23]. Their genome may be organized in either single or double-component configuration. Typically, both genomic components of bipartite geminiviruses (begomoviruses), designated DNA-A and DNA-B, are required for systemic infection. DNA-A encodes proteins involved in DNA replication (Rep and REn), transcriptional activation of viral genes (TrAP), encapsidation of the viral genome (CP) and suppression of RNAi defense functions [AC4 and TrAP; 24], [25]. The genes on DNA-B (*NSP* and *MP*) provide functions required for systemic movement of the viral genome within infected plants [reviewed in 23], [26], [27]. The nuclear shuttle protein (NSP) facilitates the intracellular trafficking of viral DNA between the nucleus and the cytoplasm, whereas MP acts as the classic viral movement protein and potentiates the cell-to-cell movement of viral DNA.

In addition to interacting with host factors required for basic compatibility functions [28], [29], [30], NSP from CaLCuV has been demonstrated to act as a virulence factor to suppress the kinase activity of transmembrane receptor NIKs [18]. The NSP binding region of NIK1 was mapped to an 80-amino acid fragment that encompasses the active loop (A-loop) and the active site of the kinase [18]. A-loops of tyrosine and serine/threonine kinases play an essential role in controlling kinase activity through the phosphorylation status of conserved tyrosine or threonine residues within this domain [reviewed in 31]. In addition to being inhibited by the viral NSP, NIKs are presumed to be involved in antiviral defense responses based on our studies showing that loss of NIK function in Arabidopsis is linked to an enhanced susceptibility phenotype to CaLCuV infection [18], [21]. Nevertheless, it remains to be determined whether the NIK kinase domain is involved in transducing a defense signal as would be expected from a receptor-like kinase-mediated signaling event. Here we further examined the NIK biochemical properties to elucidate the mechanism of receptor activation as well as its inhibition by viral NSP. We show that NIK requires kinase activation to mediate an antiviral signaling and thus is an authentic defense receptor. In addition, our data also substantiate the notion that regulated nucleocytoplasmic trafficking of rpL10 links the antiviral response to receptor activation.

## Results

### Mutation at threonine residues within the A-loop of NIK1 impacts autophosphorylation

**Figure 1:**
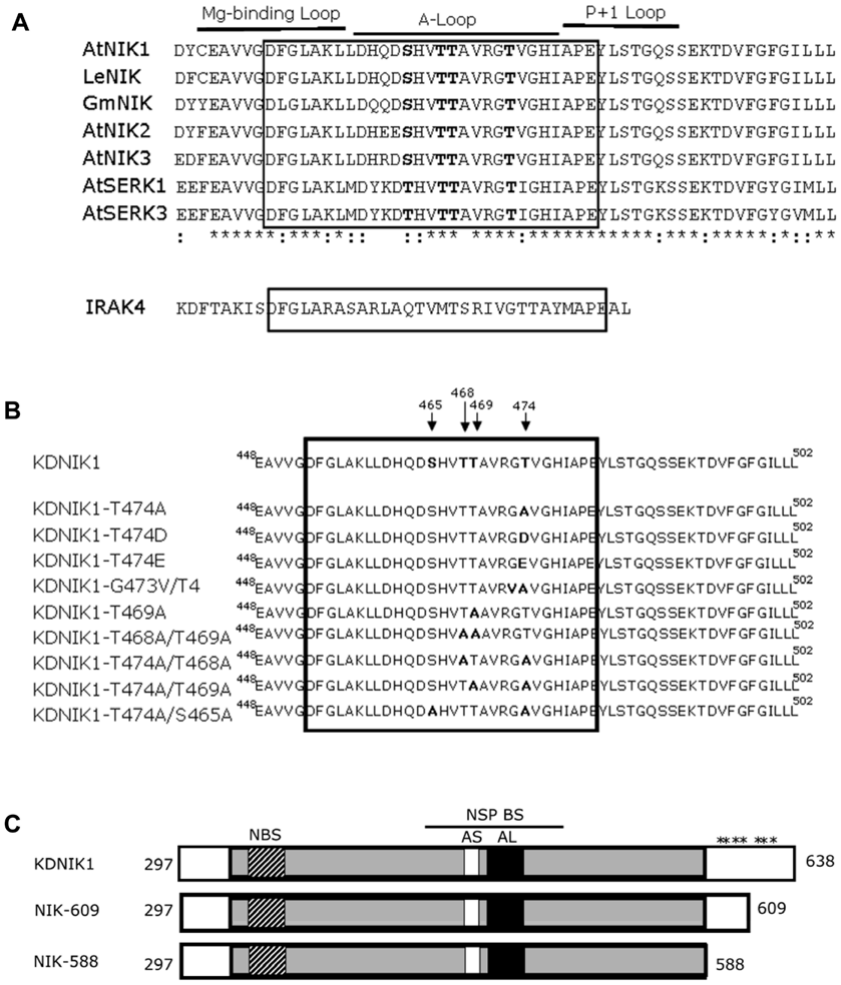
Conservation in the LRRII-RLK family and sequence position of mutant NIKs. (A). Sequence alignment of the 29 amino acid activation segment (boxed) of NIKs and other members of the LRRII-RLK family. The activation segment of NIK1 was compared to its counterparts from tomato (LeNIK) and soybean (GmNIK) and to others members of the Arabidopsis LRRII-RLK family, as indicated. Conserved residues as potential phosphorylation sites are shown in bold letters. The corresponding region of IRAK4 (30 amino acids) is shown below for comparison. (B). Schematic representation of the site-directed mutations within the NIK1 A-loop. The mutant kinases are indicated by their names and their respective mutations are indicated in bold letters. The arrows in the NIK1 sequence show mutated amino acid residues and the numbers refer to their respective positions. Boxed sequence delimits the activation segment. (C). Schematic representation of the kinase domain of two truncated forms of NIK1. The solid gray box represents the serine/threonine kinase domain; NBS represents the nucleotide binding site; AS, the active site; AL, the activation loop; NSP BS, the NSP binding site. The numbers correspond to amino acid positions on NIK1 primary structure and asterisks represent the relative position of serine residues.

**Figure 2:**
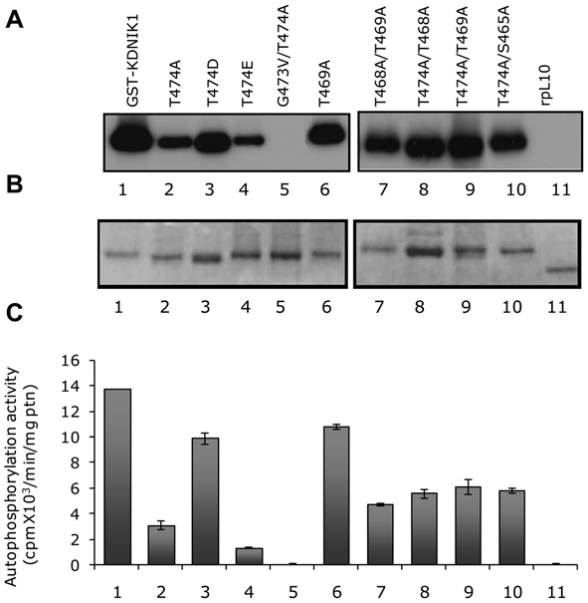
Autophosphorylation properties of mutant NIK1 proteins. GST-fusion proteins (as indicated) were incubated with [γ-^32^P]ATP, separated by SDS-PAGE and stained with Coomassie-brilliant blue. Phosphorylated proteins were visualized by autoradiography (A). The coomassie-stained gel (B) was used to normalize protein loading with Multi Gauge V3.0 (Fujifilm) software. The radioactivity incorporated into proteins was quantified by phosphoimaging (C). The relative kinase activity was expressed as Vunits/min/µg protein. Values are given as mean±S.D. of three determinations.

Activation of many kinases requires phosphorylation of the activation segment that is defined by the region delimited by two conserved tripeptide motifs, DFG and APE [31]; Figure 1A. Conserved secondary elements in this segment include the magnesium binding loop, β9, at the N-terminus, the centrally located activation loop (A-loop) and the P+1 loop at the C-terminus. The activation segment is highly conserved among members of the Arabidopsis LRRII-RLK sub-family and NIK counterparts from other plant species, such as tomato (LeNIK) and soybean (GmNIK) (Figure 1A). In the case of SERK1, the conserved Thr-462 and Thr-468 residues within the A-loop have been shown to be intermolecular targets of SERK1 kinase activity *in vitro* [32]. Likewise, the conserved SERK3/BAK1residues Thr-446, Thr-449 and Thr-455 have been shown to be phosphorylated *in vitro* as well as *in vivo* in response to brassinosteroid signaling [33]. Conservation of more distantly related kinases can also be seen by computer-assisted threading of the amino acid sequence of NIK1 onto the Ser/Thr kinase 2oidB (Interleukin 1 receptor associated kinase 4, IRAK4) as a template [34]. A striking feature is the almost perfect overlay of the activation segments of the two proteins which correspond to regions that have been shown to regulate kinase function. These observations prompted us to investigate the role of putative phosphorylation sites within NIK1 A-loop through site-directed mutagenesis (Figure 1B). The resulting mutant kinase domains were expressed as GST fusions (Figure S1) and examined for autophosphorylation activity (Figure 2). We have previously demonstrated that the kinase domain of NIK1 fused to GST exhibits Mg^2+^- dependent autophosphorylation activity that occurs intermolecularly [18].

**Figure 3:**
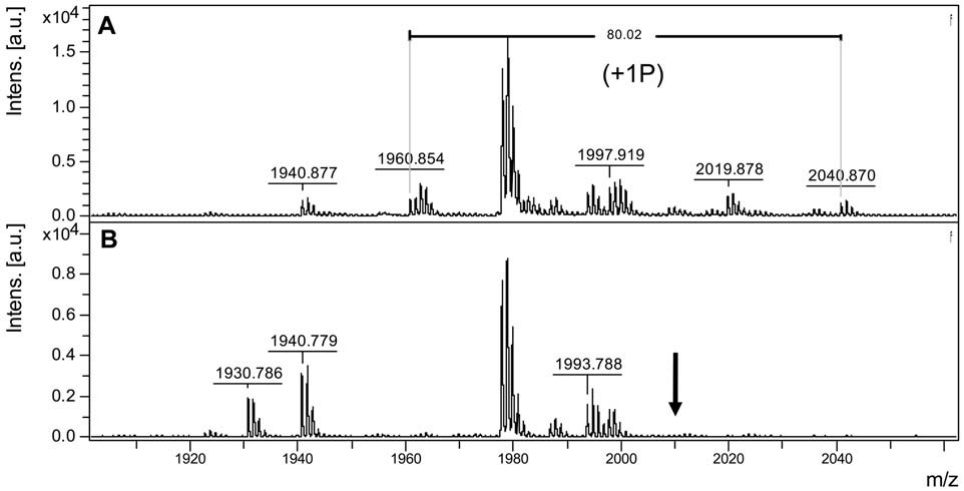
MALDI-TOF analysis of the phosphorylation state of Thr-474 in GST-KDNIK1. Purified GST-KDNIK1 (A) or GST-KDT474A (B) was autophosphorylated *in vitro*, separated by SDS-PAGE and visualized by Coomassie Blue staining. The stained bands were excised from the gel and subjected to trypsin digestion, and the resulting fragments were analyzed by MALDI-TOF mass spectroscopy. In A, among the detected molecules, peaks with *m*/*z* values of 1960.854 and 2040.870 were respectively attributed to the non- and mono-phosphorylated forms of the peptide ^473^GTVGHIAPEYLSTGQSSEK^491^. In B, the peak with *m*/*z* value of 1930.9504 corresponds to the mutated peptide ^473^GAVGHIAPEYLSTGQSSEK^491^ and the arrow indicates the absence of the predicted mono-phosphorylated form (m/z = 2010.9504) of the mutated peptide. Peptide mass was calculated *in silico* from mutated and wild-type GST-KDNIK1 sequences, using *PeptideMass* software (www.expasy.ch/). MALDI-TOF spectrum was analyzed using *FlexAnalisys* package (Bruker Daltonics). Only the regions of the spectra containing the peptides of interest are shown.

**Figure 4:**
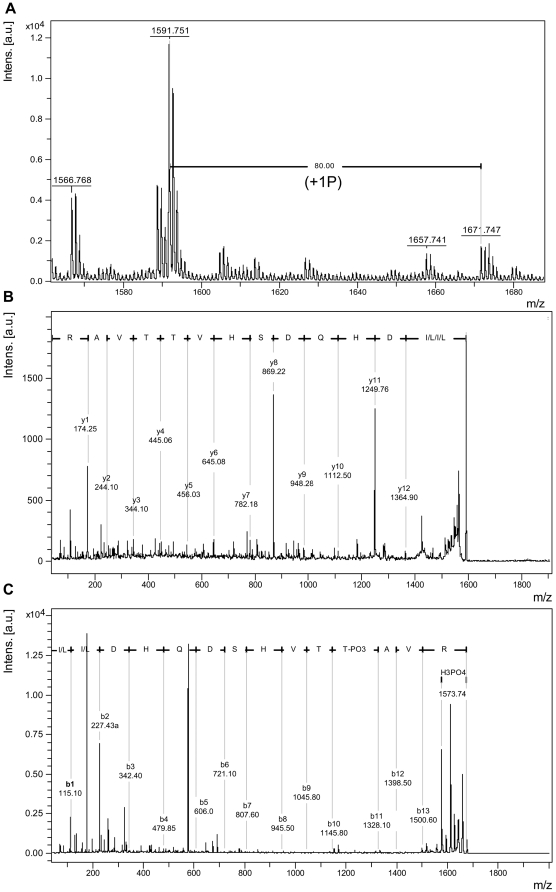
MS/MS analyses of GST-KDNIK1 reveal phosphorylation at Thr-467. (A). MALDI-TOF MS spectrum of GST-KDNIK1-derived tryptic fragments showing the ion ([M+H] 1671.74) and a mass shift of 80 Da as an indicative of phosphorylation on peptide ^459^LLDHQDSHVTTAVR^472^ ([M+H] 1591.75). (B) and (C). MS/MS spectra of the ions ([M+H] 1591.701) and ([M+H] 1671.70) that correspond to the peptides LLDHQDSVTTAVR and LLDHQDSVTTpAVR, respectively.

Replacement of Thr-474 with alanine (T474A) strongly inhibited autophosphorylation as the T474A mutant exhibited only 23% residual activity (3.1×10^3^ cpm/µg/min) as compared to NIK1 (13.7×10^3^ cpm/µg/min; Figure 2). Very likely, this effect was due to the removal of Thr and not due to the nature of the newly introduced amino acid residue because glutamate in the same position (T474E) also promoted a similar inhibition of autophosphorylation from 13.7×10^3^ to 1.32×10^3^ cpm/µg/min (lane 4). Nevertheless, replacement of Thr-474 with aspartate (T474D) did not promote an accentuated impact on autophosphorylation and the T474D mutant retained 72% activity (9.9×10^3^ cpm/µg/min; Figure 2C, lane 3). This slight effect on the relative activity of the mutant kinase may be due to the loss of an incorporated radioactive phosphate rather than a decrease in the autophosphorylation activity. We confirmed that the Thr-474 residue can be *in vitro* phosphorylated by performing matrix-assisted laser desorption ionization time-of-flight (MALDI-TOF) mass spectrometric analyses of tryptic digests of GST-KDNIK1 and GST-KDT474A mutant (Figure 3). MALDI-TOF analyses of *in vitro* phosphorylated GST-KDNIK1-derived tryptic fragments revealed a peak at m/z of 1960.854 corresponding to the peptide ^473^GTVGHIAPEYLSTGQSSEK^491^ and a peak of m/z 2040.870 representing a shift of 80.02 that corresponds to the addition of a phosphate moiety on residues 473–491. To determine whether the Thr-474 was the site of phosphate incorporation, the tryptic peptides of *in vitro* phosphorylated GST-KDT474A mutant were also analyzed by MALDI-TOF mass spectroscopy. The absence of the ion 2010.9504, corresponding to 1930.9504 Da (^473^GAVGHIAPEYLSTGQSSEK^491^) +80.00 Da (see arrow in Figure 3B), is indicative that the mutated peptide was not phosphorylated, confirming that the Thr-474 residue represents the phosphorylation site on wild type peptide ^473^G-K^491^. Taken together, these results indicate that phosphorylation of the Thr-474 residue is crucial for NIK activity, as introduction of a correctly positioned carboxylate from aspartate at this position restored autophosphorylation activity. In addition, they are suggestive that regulation of kinase activity depends on a phosphorylation-induced conformational change of the A-loop, because a mutant with glutamate (T474E) had only 9.6% residual activity. We interpret this drop as reflecting antagonism of the activation process by imposition of steric constraints to a required conformational change. In support of this model, changing the conserved Gly-473 residue in the T474A mutant to valine (G473V/T474A) totally abolished autophosphorylation activity and indicated that removing a strong structural constraint by substituting the flexible Gly-473 prevented the A-loop from adopting an active conformation. In contrast, mutation at Thr-469 caused a much lower impact on autophosphorylation activity of the resulting mutant kinase (10.9×10^3^ cpm/µg/min compared to 13.7×10^3^; Figure 2C, lane 6) and like T474D, may reflect reduction of the incorporated radioactivity without alteration of the autophosphorylation activity of the protein. In fact, using MALDI-TOF mass spectrometric analyses we detected a mono-phosphorylated form (peak at m/z of 1671.74) of the peptide ^459^LLDHQDSHVTTAVR^472^ (peak of m/z 1591.75, Figure 4A). MALDI-TOF/TOF analysis of the respective peptides was further used to examine the phosphorylation site on the monophosphorylated form. In Figure 4B, the MS/MS spectrum of the non-phosphorylated ^459^L-R^472^ peptide is shown. Phosphorylated peptides were confirmed by identification of a strong signal ([M+H] 1573,74) corresponding to the mass difference of −98.0 Da from the neutral loss of phosphoric acid (H_3_PO_4_), as indicated in Figure 4C. Phosphorylation at Thr-469 was confirmed by the presence of the ion b11 [M+H] 1328.10 that corresponds to the fragment LLDHQDSVTT-PO3.

To confirm the regulatory role of the A-loop phosphorylation, we made double mutations at T474A and the conserved putative phosphorylation sites, Ser-465, Thr-468 and Thr-469, as indicated in Figure 1B. All double mutants exhibited reduced autophosphorylation activity which varied from 34% to 40% of the normal NIK1 activity (Figure 2C). Although we did not obtain a Thr-468 single mutation, the substitution of this residue to alanine in the T469A mutant (T468A/T469A) caused a strong reduction in autophosphorylation down to 34% activity (4.7×10^3^ cpm/min/mg, lane 7) as compared to the normal kinase, suggesting that Thr-468 also plays a relevant role in the kinase activation.

### NIK1 kinase inhibition by the geminivirus NSP

**Figure 5:**
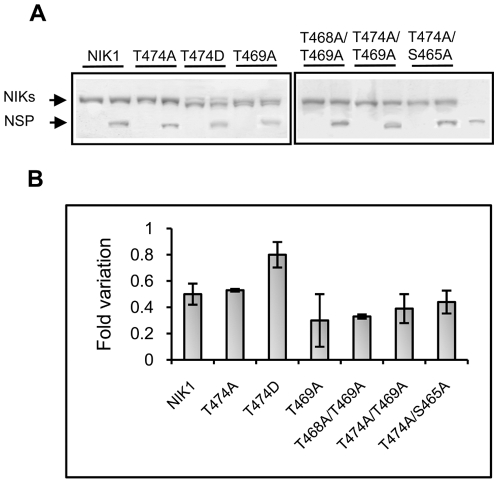
Inhibition of NIK1 autophosphorylation activity by NSP. GST-fusions (as indicated) were incubated with [γ-^32^P]ATP in the presence and absence of GST-NSP. (A) SDS-PAGE of proteins from the reaction of autophosphorylation. Proteins added to the autophosphorylation reactions were analyzed by coomassie-stained SDS-PAGE and further quantified by densitometric scanning to normalize protein levels. (B) Fold variation on autophosphorylation activity in the presence of mutated NIKs. Phosphorylated proteins in paired reactions +/− GST-NSP were quantified by scintillation counting of excised protein bands. NSP inhibition is expressed as the fold decrease of autophosphorylation activity in the presence of the viral protein.

Previous work had indicated that the NSP binding site on NIK overlaps two kinase sub domains VIb and VIII [18]. This observation raised the possibility that NSP binding to NIK might prevent A-loop transphosphorylation and hence kinase activation (Figure 1C). Inclusion of GST-NSP fusion in phosphorylation assays *in vitro* promoted a 50% reduction on NIK1 autophosphorylation (Figure 5). Except for the T474D mutant, NSP effectively caused a further 50–70% reduction in the residual activity of the mutant kinases independently of the mutation in the conserved residues of NIK1. The capacity of NSP to inhibit the mutant proteins indicates that any effect of the single and double amino acid replacements on NIK structure was too small to impair NSP binding (Figure 1C). Thus, the reduction in kinase activity by Thr replacements within the A-loop may be due to the lack of phosphorylation sites rather than a global misfolding of the kinase domain upon introduction of a different amino acid residue.

**Figure 6:**
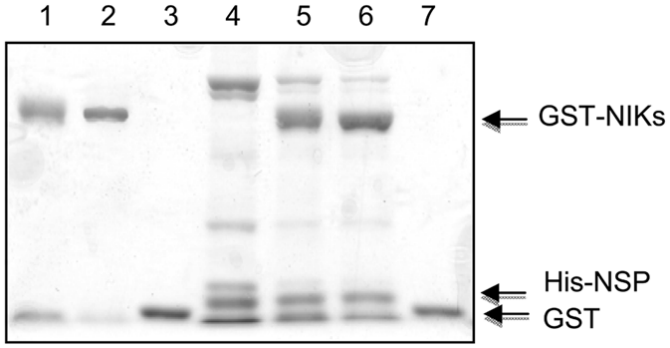
NSP interacts with T474D *in vitro.* Bacterially produced His-NSP (lane 4) was allowed to interact with GST-KDNIK1 (lane 1), GST-T474D (lane 2) or GST (lane 3) previously linked to glutathione-Sepharose beads. The retained proteins were analyzed by Coomassie-stained SDS-PAGE. Lane 6 is the result of a GST-T474D-driven pull down of NSP. GST-KDNIK1 (lane 5) and the control GST (lane 7) were also used to drive NSP pull down on glutathione-Sepharose beads protein.

In contrast to the alanine-substituted NIK A-loop mutants, the T474D mutant showed an attenuated effect of NSP on inhibition of autophosphorylation with only a 20% reduction in kinase activity in the presence of NSP. The lesser effect of NSP on inhibition of the activity of the T474D mutant could be due to a conformational change leading to destabilization of the NSP-NIK complex when Thr-474 was replaced with aspartate. To address this issue we produced NSP and T474D in *E. coli* with different tagging systems and monitored the interaction between the recombinant proteins was monitored by pull down assays (Figure 6). Bacterially expressed His-tagged NSP (lane 4) was incubated with GST-tagged NIK (lane 1), GST-T474D (lane 2) or GST alone (lane 3) and the resulting complexes were isolated on glutathione Sepharose beads. His-tagged NSP bound to GST-NIK (lane 5) and GST-T474D (lane 6) but not to GST alone (lane 7), confirming that NSP binds T474D *in vitro*. More likely, replacing Thr-474 with aspartate bypassed the inhibitory effect of NSP on Thr phosphorylation at this position. This hypothesis is consistent with an inhibitory mechanism in which the NSP-NIK complex formation modulates the phosphorylation state of the A-loop and thus kinase activation.

### Conserved threonine residues within the A-loop play distinct roles in kinase function

**Figure 7:**
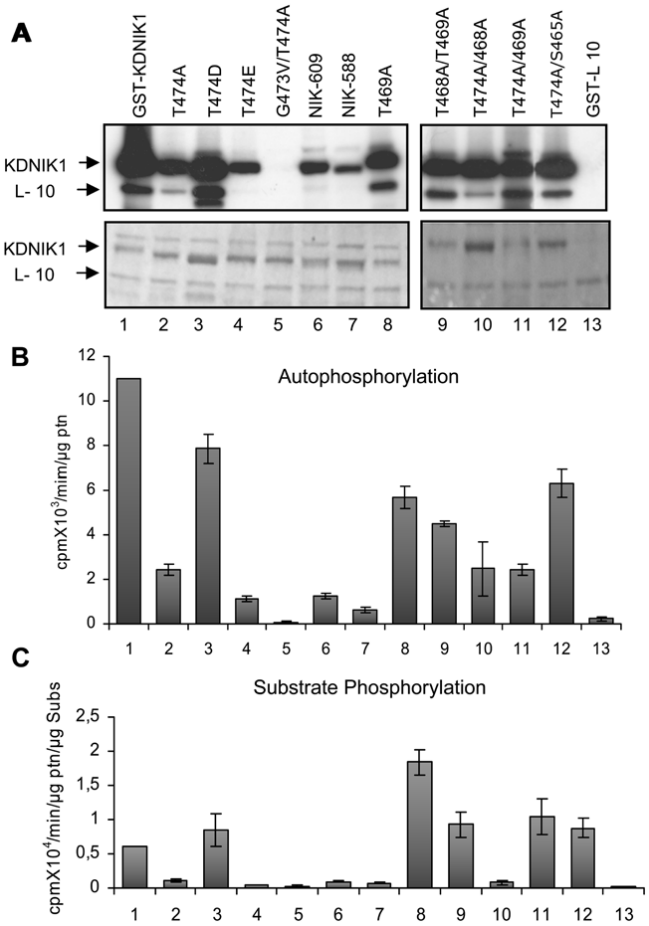
Substrate phosphorylation activity of NIK1 and mutants on rpL10. Purified GST fusions (as indicated) were incubated with equal amounts of GST-L10 in the presence of [γ-^32^P]ATP and separated by SDS-PAGE (A). The gels were stained with Coomassie-brilliant blue (bottom) and visualized by autoradiography using a phosphoimager (top). Protein loading was normalized by densitometric scanning of the Coomassie-stained gel. The relative activity of autophosphorylation (B) and phosphorylation of the substrate (C) were quantified and expressed as Vunits/µg enzyme/min and Vunits/µg enzyme/µg substrate/min, respectively.

The ribosomal protein L10 has been shown to be a substrate of NIK1 that functions as the immediate downstream component of the NIK-mediated antiviral signaling [21], [22]. Inclusion of the recombinant GST-L10 fusion in the kinase assay demonstrated that the ribosomal protein was efficiently phosphorylated by NIK1 (Figure 7). There was no measurable ^32^P incorporation into GST when GST alone was incubated with KDNIK1 and [γ^32^P]ATP (data not shown; Fontes et al., 2004). We also monitored the effect of the point mutations on the substrate phosphorylation activity of NIK1 (Figure 7). In general, mutations that negatively affect the autophosphorylation activity of the kinase protein, such as T474A, T474E, G473V/T474A, T474A/T468A, also reduced the capacity of the kinase to phosphorylate the substrate rpL10 (Figures 7B and 7C). These results are consistent with the current model for Ser/Thr kinase regulation in which autophosphorylation promotes kinase activation and precedes substrate phosphorylation [35], [36]. In contrast, replacing the essential Thr-474 residue with aspartate caused a 28% reduction in autophosphorylation activity (Figures 2 and 7B, lane 3), but promoted a 50% increase (up to 150% activity) in the substrate phosphorylation activity [Figure 7C, compare lane 1 (0.6×10^4^ cpm/µg ptn/min/µg substrate) with lane 3 (0.9×10^4^ cpm/µg ptn/min/µg substrate)]. This observation suggests that introduction of a correctly positioned carboxylate at position 474 causes constitutive activation of the protein by mimicking the induced phosphorylation of Thr-474. In addition, it indicates that phosphorylation of Thr-474 is a determinant of kinase activation. Likewise, the T469A mutant caused about 40% decrease in autophosphorylation, but exhibited a 3-fold higher substrate phosphorylation (1.83×10^4^ cpm/µg ptn/min/µg substrate) than that of the wild type kinase (Figure 7C, compare lanes 8 and 1). Very likely phosphorylation at Thr-469 inhibits substrate phosphorylation, as mutation of this residue to alanine relieves repression and enhances the capacity of the enzyme to phosphorylate rpL10. This inhibitory effect of Thr-469 was further evidenced in T468A/T469A (lane 9) and T474A/T469A (lane 11) double mutants, which exhibited enhanced substrate phosphorylation in comparison with NIK1, in spite of harboring mutations in the relevant Thr-474 or Thr-468 residues for autophosphorylation (Figures 7B and 7C). Similarly, phosphorylation at Ser-465 may inhibit substrate phosphorylation, because mutation at this residue together with mutation at the essential kinase activation residue Thr-474 resulted in a double mutant with reduced autophosphorylation but enhanced substrate phosphorylation activity (Figure 7, lane 12).

In addition to analyzing the A-loop, we analyzed the C-terminal region by introducing a stop codon at position 609 or 588 (Figure 1C). Each deletion decreased autophosphorylation activity to the same extent and totally abolished substrate phosphorylation (Figure 7, lanes 6 and 7). These results indicate that the C-terminal fragment, delimited by positions 609 to 638, may exhibit a regulatory role. Inspection of this region revealed the presence of three serine residues at positions 613, 615 and 619 with the potential to be substrates for serine/threonine kinases. Like other receptor-like serine/threonine kinases, NIK1 may possess several other autophosphorylation sites that represent docking sites for substrate recruitment or play distinct regulatory function. Although the biological relevance of this region on kinase function remains to be determined as it extends beyond the scope of the present investigation, these preliminary experiments suggest that NIK1 shares with receptor-like serine/threonine kinases the same complex mechanism of autophosphorylation-dependent activation of kinase function.

### Thr-474 and Thr-469 exhibit antagonistic roles in regulation of NIK-mediated antiviral signaling

**Figure 8:**
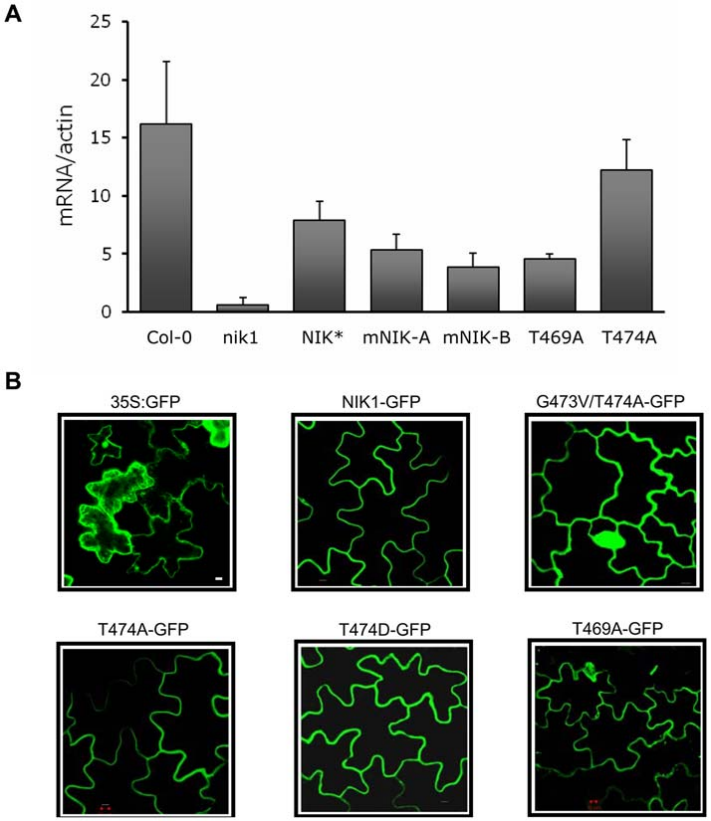
Ectopic expression of mutated *NIK1* genes. (A). Expression of intact and mutated *NIK1* genes in *nik1* KO lines. Knockout lines were stably transformed with wild type *NIK1* (NIK1*), *G473V/T474A* (mNIK1-A and mNIK1-B independently transformed lines), *T469A* or *T474A* mutant constructs and transgene expression was analyzed by real time RT-PCR. Col-0 corresponds to the expression of endogenous *NIK1* in wild type background. Values are given as mean±SD from three replicates. (B). Subcellular localization of NIK1-GFP and NIK mutant-GFP fusion proteins in *Nicotiana tabacum* leaves. Confocal fluorescence images from epidermal cells of *Nicotiana tabacum* leaves 72 hours after agroinoculation with NIK1-GFP, G473V/T474A-GFP, T474A-GFP, T474AD-GFP or T469A-GFP, under the control of the 35S promoter.

**Figure 9:**
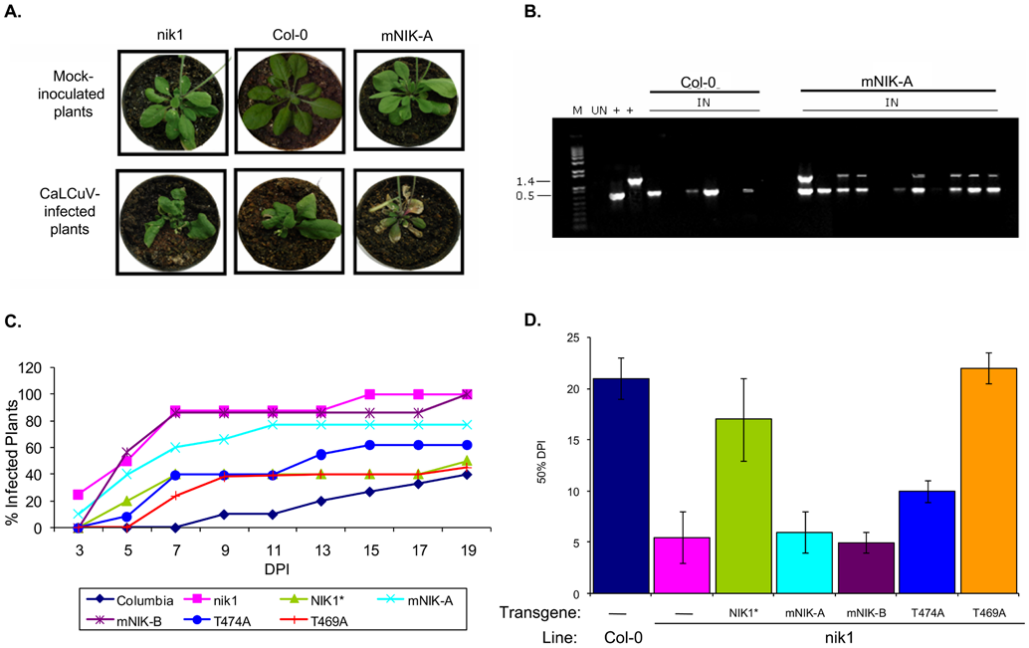
Antagonistic roles of Thr-469 and Thr-474 phosphorylation sites in NIK-mediated defense signaling. Ecotype Columbia (Col-0), nik1 lines or nik1 transgenic lines ectopically expressing wild type *NIK1* (NIK1*), *NIK1G473V/T474A* double mutant kinase gene (mNIK-A and mNIK-B independently transformed lines), *T474A* or *T469A* transgenes were inoculated with CaLCuV by biolistic delivery. (A). Symptoms associated with CaLCuV infection in transformed knockout lines. On the top, the indicated plants were bombarded with tungsten particles without viral DNA. The bottom panels show infected plants at 14 days post-inoculation (DPI). The panels show representative samples of Col-0, nik1 and mNIK-A plants. (B). Detection of viral DNA in infected lines. Total DNA was isolated from infected plants at 7 DPI and detected with viral DNA-specific primers and transgene-specific primers in the same reaction. IN refers to CaLCuV-inoculated plants and UN to mock-inoculated plants. + indicates control plasmid DNA as template. The gel shows representative samples of Col-0 and mNIK-A plants. Each lane represents individual plants. The upper band (1.4 kb) is the amplified fragment from the transgene and the lower band (0.5 kb) is the viral DNA fragment. (C). Course of infection in *nik1* transgenic lines ectopically expressing *NIK1* (NIK*), *NIK1G473V/T474A* double mutant kinase gene (mNIK-A and mNIK-B), *T469A* or *T474A* transgenes. The values represent the percentages of systemically infected plants at different DPI^50%^ and are given as mean of three determinations from independent experiments. (D). Infection rates in *nik1* transgenic lines. The infection rate is expressed as number of DPI required to get 50% infected plants (DPI 50%). The data are means of four independent experiments.

Assignment of NIK as a transmembrane signaling receptor that mediates an antiviral defense response has been based on the biochemical properties of the kinase *in vitro*, its inhibition by the geminivirus NSP and the enhanced susceptibility phenotype to geminivirus infection of knockout lines [18]. To determine whether the NIK-mediated antiviral signaling is indeed transduced through the intracellular kinase domain *in vivo*, functional complementation assays were performed by expressing the NIK1 wild type protein or a mutant NIK1 harboring an inactive kinase domain (G473A/T474A) in *nik1* null alleles. We also included the mutants T474A and T469A in the complementation assays to dissect the functional significance of these mutations *in vivo*. The transformed plants were confirmed by PCR (data not shown) and the transgene expression was monitored by real time RT-PCR for a transgenic line ectopically expressing the wild type protein (NIK*), two independently transformed KO lines expressing the G473V/T474A mutant protein (mNIK-A and mNIK-B), and transformed lines expressing T469A or T474A mutant proteins (Figure 8A). In addition, we also demonstrated by transient expression in tobacco leaves that the mutant receptors fused to GFP accumulated to detectable levels in transfected plants, as they displayed the same fluorescence pattern as a GFP-fused wild type kinase (Figure 8B). Immunoblots showed that NIK1 and mutant proteins fused to GFP accumulated to similar levels when transiently expressed in tobacco leaves [data not shown, 21]. These results confirmed that the mutations in the kinase domain of NIK1 did not affect the capacity of the protein to be associated with the cell surface and to accumulate stably in transformed plants. The transgenic lines expressing the intact NIK1 (NIK*), the double mutant protein (mNIK-A and mNIK-B), T469A and T474A mutants were biolistically inoculated with an attenuated form of CaLCuV [18]. All inoculated lines developed typical symptoms of CaLCuV infection although with different intensity (Figure 9A and data not shown). The accumulation of viral DNA was detected in all symptomatic plants by PCR with transgene-specific and viral DNA-specific multiplex primers (Figure 9B). Ectopic expression of wild type NIK1 in *nik1* KO lines restored the wild type phenotype with respect to susceptibility to geminivirus infection, as the transgenic lines developed attenuated symptoms (mild stunting with epinasty and moderate chlorosis) and displayed similar infection rates as Col-0 (Figure 9C). In other independent experiments, the infectivity data, expressed as DPI^50%^ (days post-inoculation to reach 50% of infected plants), further confirmed that NIK* and Col-0 displayed the same efficiency of virus infection (Figure 9D). In contrast, ectopic expression of the G473A/T474A inactive kinase in KO lines (mNIK-A and mNIK-B) did not complement the *nik1* loss-of-function defect and the independent transgenic lines kept a similarly enhanced susceptibility phenotype as the *nik1* line (Figures 9C and 9D). Conversely, the mutant T469A with a high substrate phosphorylation activity complemented the nik1 loss-of-function defect as efficiently as the NIK1 transgene (NIK*) and the T469A transformed line displayed an infection rate similar to NIK1* (Figure 9C), with DPI^50%^ similar to Col-0 and NIK1* (Figure 9D). Ectopic expression of T474A defective kinase did not fully complement the nik1 loss-of-function phenotype and an enhanced susceptibility phenotype in T474A lines was apparent from 12 days post-inoculation as compared to that of NIK* and T469A lines (Figure 9C). DPI^50%^ resulting from T474A expression was slightly higher than that of the inactive kinase (mNIK-A and mNIK-B) lines, but yet significantly lower than that of NIK* and T469A lines (Figure 9D). These results establish that NIK1 functions as an authentic defense receptor as it requires activation to mediate an antiviral signaling. Furthermore, they confirmed *in vivo* that phosphorylation at position Thr-474 is essential for full NIK1 kinase activation and defense signaling as opposed to phosphorylation at position Thr-469 that is likely to repress substrate phosphorylation activity.

### Thr-474 and Thr-469 residues sustain their antagonistic roles in NIK-mediated nuclear relocalization of rpL10

**Figure 10:**
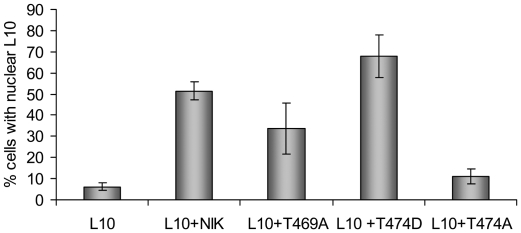
Opposed roles of Thr-469 and Thr-474 phosphorylation sites in NIK-mediated nuclear relocalization of rpL10. Tobacco leaves were co-agroinfiltrated with YFP-L10 and GFP-fusions, as indicated, and the subcellular localization of the fluorescent fusion proteins was monitored by confocal microscopy. The percentage of co-transfected cells containing YFP-L10 fluorescence over the nucleoplasm was registered. Values are the mean±SD of three determinations from independent experiments. In each experiment, a total of 100 to 150 cells were observed.

The T474D mutant was not included in our complementation assay because we could not recover T474D expressing transformed lines. However, the relevance of this mutation has been recently addressed *in vivo* by the demonstration that the T474D mutant exhibits an enhanced capacity to phosphorylate and relocate rpL10 from the cytoplasm to the nucleus when transiently expressed in tobacco infiltrated leaves [21]. NIK-mediated nuclear relocalization of rpL10 is thought to trigger a defense response that impairs virus proliferation or spread. We used the antagonistic mutants T469A and T474A to examine whether redirection of rpL10 to the nucleus would couple NIK activation to the defense response. The subcellular localization of rpL10 in co-transfected leaf cells was assayed by confocal microscopy (Figure S2, merged field) and the frequency of cells with nuclear-localized rpL10 was recorded (Figure 10). Ectopically expressed rpL10 in transfected cells is predominantly localized in the cytoplasm and only a small fraction (5%) of transfected cells contains nuclear rpL10. Co-expression with NIK1, however, altered the nucleocytoplasmic shuttling of rpL10 because the cell frequency with nucleus-localized YFP-rpL10 was significantly increased to 50.6±4% of co-transfected leaf cells. As expected [21], the hyperactive T474D mutant enhanced translocation of rpL10 to the nucleus (68±8% of transfected cells with nuclear rpL10). While the inactive G473V/T474A mutant [21] or the defective T474A mutant failed to redirect rpL10 to the nuclei of co-transfected cells, the T469A mutant was almost as effective as wild type NIK1 in mediating rpL10 nuclear translocation as it increased the frequency of co-transfected cells with nuclear rpL10 to 35±13%. Although T469A did not enhance translocation of rpL10 to the nucleus as would be expected from its increased *in vitro* substrate phosphorylation activity when compared with wild type NIK1, these results showed that mutation at Thr-469 did not impair NIK-mediated nuclear relocation of rpL10, confirming *in vivo* that the Thr-469 and Thr-474 phosphorylation sites play distinct roles in NIK activation. They also demonstrated that mutations at phosphorylation sites within the A-loop of NIK1 that antagonistically regulate kinase function acted similarly in conferring tolerance to virus infection and nuclear relocalization of rpL10. This positive correlation is consistent with the notion that the regulated nucleocytoplasmic shuttling of rpL10 links the antiviral response to receptor activation.

## Discussion

The NIK receptor has been hypothesized to mediate an antiviral defense response through a reversible phosphorylation strategy that both initiates a signaling pathway as well as modulates the consequent adaptive response. Here we separated auto- and substrate phosphorylation and identified Thr-474 as targets for intermolecular autophosphorylation and full kinase activity. Conversely, a Thr-469 residue within the A-loop seems to play an inhibitory role in kinase function as its individual replacement with alanine did not impair autophosphorylation but enhanced substrate phosphorylation.

The Thr-468 and Thr-474 residues aligned at the same position as Thr-462 and Thr-468 on SERK1 as well as Thr-449 and Thr-455 on SERK3/BAK1, other members of the LRRII-RLK sub-family. Both threonine residues within SERK1 A-loop and BAK1 A-loop have been shown to play relevant roles in kinase function. Thr-468 on SERK1 is absolutely essential for *in vitro* kinase activity [33] and the equivalent residue on BAK1, Thr-455, has been shown to play a critical role in BAK1 signaling [34]. Our results implicate Thr-468 and Thr-474 on NIK1 as functional analogs of Thr-462 and Thr-468 on SERK1, as well as of Thr-449 and Thr-455 on SERK3/BAK1, respectively. Individual mutations in NIK1 respective residues promote an 80% reduction in kinase autophosphorylation that in turn leads to equivalent decrease in the substrate phosphorylation activity. Phosphorylation of residues within the A-loop constitutes one of the key regulatory mechanisms not only of Ser/Thr kinases but also of Tyr kinases [35], [36], [37], [38], [39].

The four lines of evidence presented here demonstrate that the underlying mechanism for Thr-474-dependent kinase activation is due to phosphorylation. Firstly, MALDI-TOF mass spectrometric analyses of tryptic digests of *in vitro* phosphorylated GST-KDNIK1 and GST-KDT474A revealed that Thr-474 was the phosphorylated residue on the tryptic phosphopeptide ^473^GTpVGHIAPEYLSTGQSSEK^491^. Secondly, replacing Thr-474 with either alanine or glutamate reduced kinase activity to similar extent, eliminating the possibility that the variation observed might have been due to a possible effect of the newly introduced amino acid residue on structure. Thirdly, the T474A mutant keeps the capacity to be further inhibited by NSP, indicating that replacement of Thr-474 with alanine did not cause a global misfolding of the kinase domain that would impair NSP binding and affect kinase activity. Finally, the introduction of a negatively charged aspartate residue at position 474 did not alter the autophosphorylation activity but enhanced the efficiency of substrate phosphorylation. The findings extend genetic studies linking inactivation of NIK genes to enhanced susceptibility to geminiviruses and protein structural analysis of residues required for kinase activity. Very likely the presence of a correctly positioned carboxylate mimicked the phosphorylation state of Thr-474 resulting in constitutive activation of the receptor kinase. SNF-1 kinases and plant SOS2 (Salt Overlay Sensitive2)-like protein kinases – PSKs have been shown to be highly activated by substitution of phosphoryalated residues with aspartate within the activation loop [40], [41], [42]. However, the extent by which A-loop phosphorylation-induced kinases are activated by mutations of phosphorylation residues to aspartate or glutamate has been shown to vary considerably among Tyr kinases and Ser/Thr kinases [reviewed in 43]. In the case of NIK1, we further demonstrated that the correct position of the introduced carboxylate within the A-loop was relevant for activity because there was almost no detectable activity with the T474E mutant. The contrasting results of T474D and T47E on NIK1 activity resemble those found for the A-loop mutants of Protein Kinase C, in which the replacement of its critical Thr-500 residue with glutamate increases kinase activity, whereas aspartate in same position inactivates kinase [44]. While the extra methylene in glutamate may be necessary to position the carboxyl group in the correct orientation for electrostatic interactions that could align catalytic residues in the protein kinase C activation loop, it clearly imposes a conformational constraint for NIK1 activation. Similar results have been reported for the SERK1 protein in which the substitution of Thr-468 residue, which is analogous to the NIK1 Thr-474 residue, with glutamate abolishes autophosphorylation activity [33]. Taken together, our results with the Thr-474 mutants of NIK1 are consistent with the current activation model of kinases in which phosphorylation of the A-loop induces conformational changes to position correctly the residues that interact directly with the substrate and the catalytic domain [reviewed in 31]. In addition they implicate the NIK1 residue Thr-474 as the critical phosphorylation site for kinase activation.

We also demonstrated that although Thr-469 within the A-loop is not important for autophosphorylation it functionally antagonizes Thr-474 by playing an inhibitory role in the substrate phosphorylation activity. In fact, replacing Thr-469 with an alanine residue relieves repression and enhances considerably substrate phosphorylation. One possible explanation is that phosphorylation of these residues would antagonize phosphorylation of the relevant Thr-474 residue by blocking the conformational change that permits unrestricted access of ATP and protein substrates to the kinase active site, as that deduced from crystal structures of phosphorylated active kinases [35], [36]. The slight decrease in ^32^P incorporation into T469A upon autophosphorylation may account for loss of a phosphorylation site in the mutant proteins, favoring the argument that Thr-469 is a target of NIK1 kinase activity. In support of this, MALDI-TOF/TOF experiments conducted on the phosphorylated form of the tryptic peptide ^459^LLDHQDSHVTTAVR^472^ demonstrated that Thr-469 can be phosphorylated. However, our data do not rule out the possibility that the putative relief of structural constraints was due to the introduction of alanine at the structurally restrictive position. The confirmation that this conserved threonine residue is phosphorylated *in vivo* is necessary to distinguish between these possibilities.

The previous assignment of *NIK* as a defense gene was based on circumstantial evidence that linked the enhanced susceptibility phenotype to geminivirus infection to the loss of *NIK* function. Here we performed complementation assays in *nik1* null alleles, which demonstrated that NIK is an authentic defense signal transducer as it requires kinase activation to mediate an adaptive response to geminivirus infection. In fact, while the ectopic expression of *NIK1* in the null allele background restored the wild type phenotype by decreasing virus susceptibility, the expression of the inactive G473V/T474A kinase did not reverse the *nik1* enhanced susceptibility phenotype, which was associated with a high infection rate and development of severe symptoms upon infection. By examining the effects of the phosphorylation site mutations on NIK-mediated nuclear relocalization of rpL10, we found a perfect correlation on the ability of the mutations to affect tolerance and rpL10 nuclear relocation. The T474A mutant failed to restore the NIK1-mediated tolerance against geminivirus and to redirect rpL10 to nuclei of co-transfected cells. In contrast, mutation at Thr-469 did not impair NIK-mediated nuclear relocation of rpL10 or tolerance against geminivirus. Collectively, these results further substantiate the notion that relocation of rpL10 to the nucleus links the antiviral response to receptor activation. Furthermore, they confirmed *in vivo* the antagonistic role of the Thr-469 and Thr-474 phosphorylation sites in the regulation of NIK activation. They also suggest that the NIK1 residue Thr-474 is functionally equivalent to the corresponding BAK1 residue Thr-455 and BRI1 residue Thr-1049, which aligned at the same position in their respective A-loop and have been shown to be required for kinase function and signaling *in planta* [34], [45].

There is no precedent in the literature for negative regulation of kinase activity through phosphorylation of conserved sites within the A-loop of plant RLKs. Although phosphorylation of threonine residues within the A-loop has been demonstrated *in vitro* for SERK1 and *in vivo* for BRI1 and BAK1, the functionally relevant phosphorylated residues regulate positively kinase activity [33], [34], [45]. Our results may have uncovered a novel scheme for kinase regulation in which autophosphorylation of Thr-469 within the NIK1 A-loop would control negatively the phosphorylation of rpL10 and hence would allow the kinase to control more efficiently the extent of the response in a sustained signaling pathway. Whether this inhibitory mechanism is specific for the rpL10 substrate, providing NIK1 with the capacity to differentially phosphorylate pathway components remains to be determined. In the case of BAK1, the activation-loop residue Thr-450, which is equivalent to NIK1 Thr-469, has been shown to play independent and separate roles in BR signaling and flagellin signaling [34].

Based on the present findings and common features of LRR-RLK, a mechanistic model for a NIK-mediated defense signaling pathway and its interaction with the geminivirus NSP can be proposed. In this model, upon unidentified stimuli, the LRR extracellular domain undergoes oligomerization that, in turn, brings the intracellular kinase domains into close proximity, allowing them to transphosphorylate and to activate one another. The active kinase recruits and phosphorylates the downstream component rpL10 to propagate the defense-signaling cascade that impairs virus replication and/or movement. Regulation of kinase activity may be dictated by two components: a conserved Ser/Thr kinase activation component that results from autophosphorylation at Thr-474 and a novel inhibitory component at a distinct residue (Thr-469) within the A-loop that down regulates substrate phosphorylation. Counteracting the pathway proactivation mechanism, binding of NSP to the kinase domain promotes steric constraints that impair intermolecular phosphorylation at Thr-474 within the NIK A-loop. The argument that NSP acts upstream of Thr-474 autophosphorylation is supported by the finding that the hyperactive T474D mutant, in which the introduced carboxylate may mimic the phosphorylated state of Thr-474, keeps the capacity to form a stable complex with NSP *in vitro* but it is not inhibited by the viral protein. This molecular mechanism for NSP inhibition of NIK1 prevents activation of the NIK-mediated signaling pathway creating an intracellular environment that is more favorable to virus proliferation and spread.

In summary, the relevance of the present study is two-fold. Firstly, it uncovers a novel scheme for receptor serine/threonine kinase activation with different roles for distinct conserved threonine residues within the A-loop of the kinase domain. The positive regulatory role of the Thr-474 phosphorylation site promotes kinase activation triggering the antiviral response, whereas the inhibitory role of Thr-469 on substrate phosphorylation would allow the kinase to control more efficiently the extent of the response in a sustained signaling pathway. Secondly, it provides further evidence that regulated nucleocytoplasmic trafficking of rpL10 couples activation of the receptor NIK with an antiviral response. In fact, mutations at conserved threonine residues that antagonistically regulate kinase activation affect similarly the NIK capacity to elicit an antiviral response and to mediate a phosphorylation-dependent nuclear relocalization of the rpL10 downstream component.

## Materials and Methods

### DNA constructs and generation of point mutants

The NIK1 activation loop mutations were obtained through the Gene Tailor™ Site-directed Mutagenesis system (Invitrogen Life Technologies, Inc.) using the recombinant plasmid pDON-AtNIK1 [18] as template and partially overlapped primers (Table S1) that generated the nucleotide changes: Thr474 to Ala474 (pDON-NIK1T474A), or Glu474 (pDON-NIK1T474E) or Asp474 (pDON-NIK1T474D), Thr469 to Ala469 (pDON-NIK1T469A) as well as Gly473/Thr474 to Val473/Ala474 (pDON-NIK1G473VT474A). The resulting clones harboring single mutations were used to generate the double mutants: pDON-NIK1T468A/T469A, pDON-NIK1T474A/T468A, pDON-NIK1T474A/T469A, and pDON-NIK1T474A/S465A with the primers shown in Table S1. To create plasmids for *E. coli* expression, the mutant NIK1 C-terminal kinase domains (KD, encoding amino acids 297–638) were amplified from the appropriate mutated clone with the primers NBSAtNIK1F-GA (5′-aaaaagcaggcttcacaatgaggagatttggtttc-3′) and KDAtNIK1RG (5′-agaaagctgggtctcatctaggaccagagagctc-3′), introduced by recombination into the entry vector pDONR201 and then transferred to the bacterial expression vector pDEST15, resulting in GST fused to mutant kinase domains, such as pGST-KDNIK1T474A, pGST-KDNIK1T474D, pGST-KDNIK1T474E, pGST-KDNIK1G473V/T474A, pGST-KDNIK1T469A, pGST-KDNIK1T468A/T469A, pGST-KDNIK1T474A/T468A, pGST-KDNIK1T474A/T469A and pGST- KDNIK1T474A/S465A. The double mutant NIK1G473V/T474A cDNA, as well as T469A, T474A and T474D mutant cDNAs were also amplified from the respective mutated clones with the primers AtNIK1FG (5′-aaaaagcaggcttcacaatggagagtactattgtt-3′) and AtNIK1RGNS (5′-agaaagctgggtctctaggaccagagagctccat-3′), re-introduced by recombination into the entry vector pDONR201 and then transferred to the binary vector pK7FWG2 [46] to yield pK7F-NIK1G473VT474A, pK7F-NIK1T469A, pK7F-NIK1T474A and pK7F-NIK1T474D. These resulting clones harbor a GFP gene fused in-frame after the last codon of the respective mutant cDNAs under the control of the CaMV 35S promoter

A ribosomal L10 cDNA was isolated from an Arabidopsis cDNA two-hybrid library by its capacity of interacting with the kinase domain of NIK1 [22]. The L10 cDNA was amplified by PCR with appropriate extensions provided by the primers rpL10Fwd (5′-aaaaagcaggcttcacaatgggaagaagacctgt-3′) and rpL10Rvs (5′-agaaagctgggtctcagtagtgggctggcaaaaa-3′), introduced by recombination into the entry vector pDONR201 and then transferred to pDEST15 to generate GST-fused L10 (pGST-L10). To obtain the YFP gene fused before the first codon of L10, the respective cDNA was transferred from pDONR207 to 35S-YFP-casseteA-Nos-pCAMBIA1300, yielding pYFP-L10 [21]. The plasmid pGST-NSP, harboring the CaLCuV NSP sequence fused to GST, has been described previously [18].

### Expression in E. coli and purification of GST-fused proteins

*E. coli*, strain BL21::DE3 pLysS, was transformed with plasmids containing different fusions and the synthesis of the recombinant proteins was induced with 0.4 mM isopropyl-dithiogalactopyranoside (IPTG) for 16 h at 20°C and 200 rpm. The accumulation of recombinant proteins was monitored by SDS–PAGE in whole cell extracts, as well as in soluble and insoluble fractions. Cells were pelleted by centrifugation, resuspended in lysis buffer [140 mM NaCl, 2.7 mM KCl, 10 mM Na2HPO4, 1.8 mM KH2PO4, pH 7.4, 0.4% (v/v) Triton X-100 supplemented with 0.15 mg/mL of lysozyme, 0.8 mM PMSF, 1 mM benzamidine, 1 mM thiourea], incubated at 4°C for 30 min, disrupted by sonication, and centrifuged at 14,000 g for 20 min at 4°C. The GST fusions were affinity-purified using GST-Sepharose beads (GE healthcare), according to manufacturer's instructions. The efficiency of protein purification was monitored by SDS-PAGE.

### Protein kinase assay

The purified GST-fused proteins (KDNIK1T474A, KDNIK1T474D, KDNIK1T474E, KDNIK1G473V/T474A, KDNIK1T469A, KDNIK1T468A/T469A, KDNIK1T474A/T468A, KDNIK1T474A/T469A, KDNIK1T474A/S465A) were incubated alone or with the substrate GST-L10 at 25°C for 45 min in 20 µL of kinase buffer containing 18 mM HEPES pH 7.4, 10 mM MgCl_2_, 10 mM MnSO_4_, 1 mM DTT, 10 µM ATP and 5–10 µCi [γ-^32^P]ATP (3000 Ci/mmol). We also performed *in vitro* kinase assay under the same conditions as described for SERK1 mutants [33]. Phosphoproteins were resolved by SDS-PAGE. The gel was stained with Coomassie brilliant blue to verify protein loading, dried, and subjected to autoradiography. Incorporated radioactivity in protein bands was quantified by phosphoimaging and protein loading by densitometry using the Multi Gauge V3.0 software (Fujifilm). Alternatively, the protein bands were excised from the dried gel, dissolved in 30% (v/v) H_2_O_2_ and the radioactivity incorporated was measured by scintillation counting.

### MALDI-TOF/TOF analyses

For MS/MS analyses, GST-fused proteins (KDNIK1, KDT474A) were phosphorylated by incubating 10–25 µg of the purified recombinant protein at 25°C for 75 min in 20 µL of kinase buffer containing 18 mM HEPES pH 7.4, 10 mM MgCl_2_, 10 mM MnSO_4_, 1 mM DTT, 200 µM ATP. Phosphoproteins were resolved by SDS-PAGE. GST-KDNIK1 or GST-KDT474A spots were excised from SDS-PAGE gel, rinsed with distilled water and destained twice with 30-min washes in 50 mM NH_4_HCO_3_/50% acetonitrile. After dehydration in 100% acetonitrile, the gel pieces were incubated with 30 µl of 12.5 ng/µl sequencing grade trypsin (Promega, Madison, WI) for 12 h at 37°C. Tryptic peptides were extracted from the gel pieces twice with 15 µl of 1% formic acid/50% acetonitrile for 20 min. The pooled extracts were dried in speed-vac and tryptic digests were resuspended in 50% acetonitrile/1% phosphoric acid/1% TFA.

MALDI-TOF/TOF experiments were conducted on an Ultra-flex MALDI-TOF/TOF Analyzer (Bruker Daltonics). Peptides from each spot were mixed 1∶1 with matrix solution [2,5-dihydroxybenzoic acid (2,5-DHB) in 50% acetonitrile/0.1% trifluoroacetic acid/1% phosphoric acid] and 1 µl were applied to wells of a sample target plate. Peptide mass fingerprints were obtained using the reflector and positive ion mode. Mass spectra were collected from the sum of 200–800 laser shots, and monoisotopic peaks were obtained. LIFT mass spectra were acquired and metastable fragmentation was induced without the further use of collision gas.

Peptides mass spectra were matched *in silico* against the GST-KDNIK1 theoretical tryptic mass using the PIUMS software with mass tolerance of 100 ppm, one missed trypsin cleavage, fixed modification of carbamidomethyl cysteine, and variable modifications of methionine oxidation and threonine, tyrosine and serine phosphorylation. Peptides sequencing was made by visual inspection of mass spectrum using FlexAnalysis software. Phosphorylated peptides were confirmed by an increase in mass of 80 Da and a decrease of 98.0 Da corresponding to the addition of PO3 and neutral loss of H3PO4, respectively.

### RT-PCR and qRT-PCR analyses

Total RNA was extracted from Arabidopsis seedlings using TRIzol (Invitrogen). Reverse transcription (RT)-PCR assays were performed with 2 µg of total RNA, 0.5 µM of poly-dT and 1U of M-MLV reverse transcriptase (Invitrogen Life Technologies, Inc.), as previously described [47], [48]. PCR was carried out with NIK1-specific primers (AtNIK1-Fwd, 5′–accgcgatgtcaaagcagcg–3′ and AtNIK1-Rvs, 5′- atgtgacccaccgtgcctct –3′) and with actin-specific primers (AtACTIN-Fwd, 5′–atgtcgtgagccatcctgtc–3′ and AtACTIN-Rvs, 5′–acaccggattcgtgcggcat–3′) to assess the quantity and quality of the cDNA. The PCR comprised 30 cycles of 45 s at 94°C, 30 s at 55°C, and 2 min at 72°C.

Real-time RT-PCR reactions were performed on an ABI7500 instrument (Applied Biosystems, Foster City, CA), using SYBR® Green PCR Master Mix (Applied Biosystems), as described [49], [50]. The amplification reactions were performed as follows: 2 min at 50°C, 10 min at 95°C, and 40 cycles of 94°C for 15 sec and 60°C for 1 min. To confirm quality and primer specificity, we verified the size of amplification products after electrophoresis through a 1.5% agarose gel, and analyzed the Tm (melting temperature) of amplification products in a dissociation curve, performed by the ABI7500 instrument. The RNA actin was used as endogenous control to normalize all values in the real-time RT-PCR assays [49]. Gene expression was quantified using the 2^ΔC^T method.

### Plant material, growth conditions, and plant transformation

The Columbia (Col-0) ecotype of Arabidopsis thaliana was used as the wild type control and nik1 knockout line [18] was used for complementation experiments. Seeds were surface sterilized and cold treated at 4°C for 2 days in the dark and then exposed to white light. Seedlings were grown at 22°C on plates containing Murashige-Skoog medium for 3 weeks and then transferred to soil. Plants were grown in a growth chamber at 22°C under long-day conditions (16 h light/8 h dark). *nik1* KO lines were transformed with pK7F-NIK1 [18], pK7F-NIK1G473VT474A, pK7F-NIK1T469A or pK7F-NIK1T474A using the floral dip method [51]. Transgene incorporation was monitored by PCR using a forward 35S promoter-specific primer, MC36 (5′-tccttcgcaagacccttcctc-3′), and a reverse NIK1-specific primer, LRAtNIK1RG (5′-agaaagctgggtcgagtcataagagatttcgatg- 3′). Transgene expression was confirmed by real time PCR with the primers AtNIK1-Fwd and AtNIK1-Rvs. For quantitation of gene expression, we used actin as a control gene and the primers AtACTIN-Fwd and AtACTIN-Rvs, as described above. We selected two independently transformed lines expressing the NIK1G473VT474A transgene (mNIK-A and mNIK-B), one *nik1* line expressing NIK1 (NIK1*), T474A or T469A transgene for the infection assays.

### Accumulation of NIK mutant proteins *in planta*

*Nicotiana tabacunn* leaves were agroinoculated with pK7F-NIK1 [18], pK7F-NIK1G473V/T474A, pK7F-NIK1T469A, pK7F-NIK1T474A and pK7F-NIK1T474D using *Agrobacterium tumefaciens* strain GV3101, as previously described [28]. About 72 hours pos-agroinfiltration, 1-cm^2^ leaf explants were excised and fluorescence patterns were examined in epidermal cells using a Zeiss inverted LSM510 META laser scanning microscope with an argon laser and a *helium néon laser* with 40× or 60× oil immersion objective. For imaging GFP, the 488 nm excitation line and the 500 to 530 nm band pass filter were used. The pinhole was usually set to give a 1 to 1.5 µm optical slice. Post-acquisition image processing was done using the LSM 5 Browser software (Carl-Zeiss) and Adobe Photoshop (Adobe Systems).

### CaLCuV inoculation and analysis of infected plants

*Arabidopsis thaliana* plants at the seven-leaf stage were inoculated with plasmids containing partial tandem repeats of CaLCuV DNA-A and DNA-B by biolistic delivery [52] and the course of infection was monitored as described previously [18], [30]. We used an attenuated form of the virus, in which the coat protein ORF in CaLCuV DNA-A was interrupted by introducing a stop codon at amino acid position 47. The inoculated plants were transferred to growth chamber and examined for symptom development (leaf necrosis, chlorosis, leaf epinasty, leaf curly, young leaf death and stunted growth). Total nucleic acid was extracted from systemically infected leaves and viral DNA was detected by PCR with DNA-A or DNA-B begomovirus-specific primers [53]. In multiplex PCRs the stably incorporated transgene was also examined using the transgene-specific primers (MC36 and LRAtNIK1RG) in combination with DNA-B CaLCuV-specific primers (566CLCVBFBR1v, 5′-ggcgtggggtatcttactc-3′ and 1253CLCVBRBR1c, 5′-gacatagcatcggacatcc-3′). In each experiment, 20 plants of each line (Col-0, *nik1*, *nik-1* expressing NIK1 mutant proteins) were inoculated with 2 µg of tandemly repeated DNA-A plus DNA-B per plant. The course of infection was registered with data from three independent experiments. DP1^50%^ (days post- inoculation to reach 50% of infected plants) was obtained with data from four independent experiments.

### Subcellular localization of proteins

For subcellular localization of proteins, *Nicotiana tabacum* leaves were agroinoculated with pK7F-L10 or pYFP-L10 in combination with pK7F-NIK1, pK7F-NIK1T474D, pK7F-NIK1T474A, pK7F-NIK1T469A or pK7F-NIK1G473V/T474A using *Agrobacterium tumefaciens* strain GV3101. *Nicotiana tabacum* plants were grown in a greenhouse with natural day length illumination. For experimental use, plants (about three week after germination) were transferred to a growth chamber at 21°C with a 16-hour light and 8-hour dark cycle. Agrobacterium-mediated transient expression in tobacco leaf epidermal cells was conducted as previously described [21]. About 72 hours post-agroinfiltration, 1-cm^2^ leaf explants were excised and GFP and YFP fluorescence patterns were examined in epidermal cells with 40× or 60× oil immersion objective and a Zeiss inverted LSM510 META laser scanning microscope equipped with an argon laser and a helium laser as excitation source. For imaging GFP, the 458–488 nm excitation line and the 500 to 530 nm band pass filter were used. Excitation of YFP was at 514–560 nm and YFP emission was detected by using a 560–600 nm filter. Controls were performed to ensure clear separation of GFP and YFP signals. The pinhole was usually set to give a 1 to 1.5 µm optical slice. In each independent experiment, a total of 100 to 150 cells were observed and the number of cells with nucleus-localized rpL10 was recorded.

## Supporting Information

Figure S1 SDS-PAGE of E. coli- produced GST fusions. GST-fused to the C-terminal kinase domain of normal NIK1 (GST-KDNIK1) or to mutant NIK1s, as indicated, were produced in E. coli, affinity-purified, separated by SDS/PAGE and stained with coomassie brilliant blue. GST-L10 corresponds to a ribosomal protein L10 (rpL10) fused to GST and GST-NSP is a CaLCuV NSP fusion. Molecular mass markers (kDa) are shown on the left.
(1.05 MB TIF)

Figure S2 Effect of ectopic expression of NIK1 and A-loop mutants on nucleocytoplasmic shuttling of rpL10A. NIK1-GFP+YFP-L10, T469A-GFP+YFP-L10, T474D-GFP+YFP-L10 or T474A-GFP+YFP-L10 were co-expressed in tobacco leaf epidermal cells and the subcellular localization of the fluorescent fusion proteins was monitored by confocal microscopy. The frequency of co-transfected cells (merged field) with rpL10A localized within the nuclei was obtained. In each experiment, a total of 100 to 150 cells were observed. Full arrows indicate fluorescent nuclei. Scale bars are 10 µm
(5.15 MB TIF)

Table S1 Primers for mutagenesis within the kinase domain of NIK1
(0.04 MB DOC)
