# Supplementary figures and images for: Conserved Threonine Residues within the A-Loop of the Receptor NIK Differentially Regulate the Kinase Function Required for Antiviral Signaling
Source: PLoS One. 2009 Jun 3;4(6):e5781. doi: 10.1371/journal.pone.0005781 (PMC2686266; doi:10.1371/journal.pone.0005781)

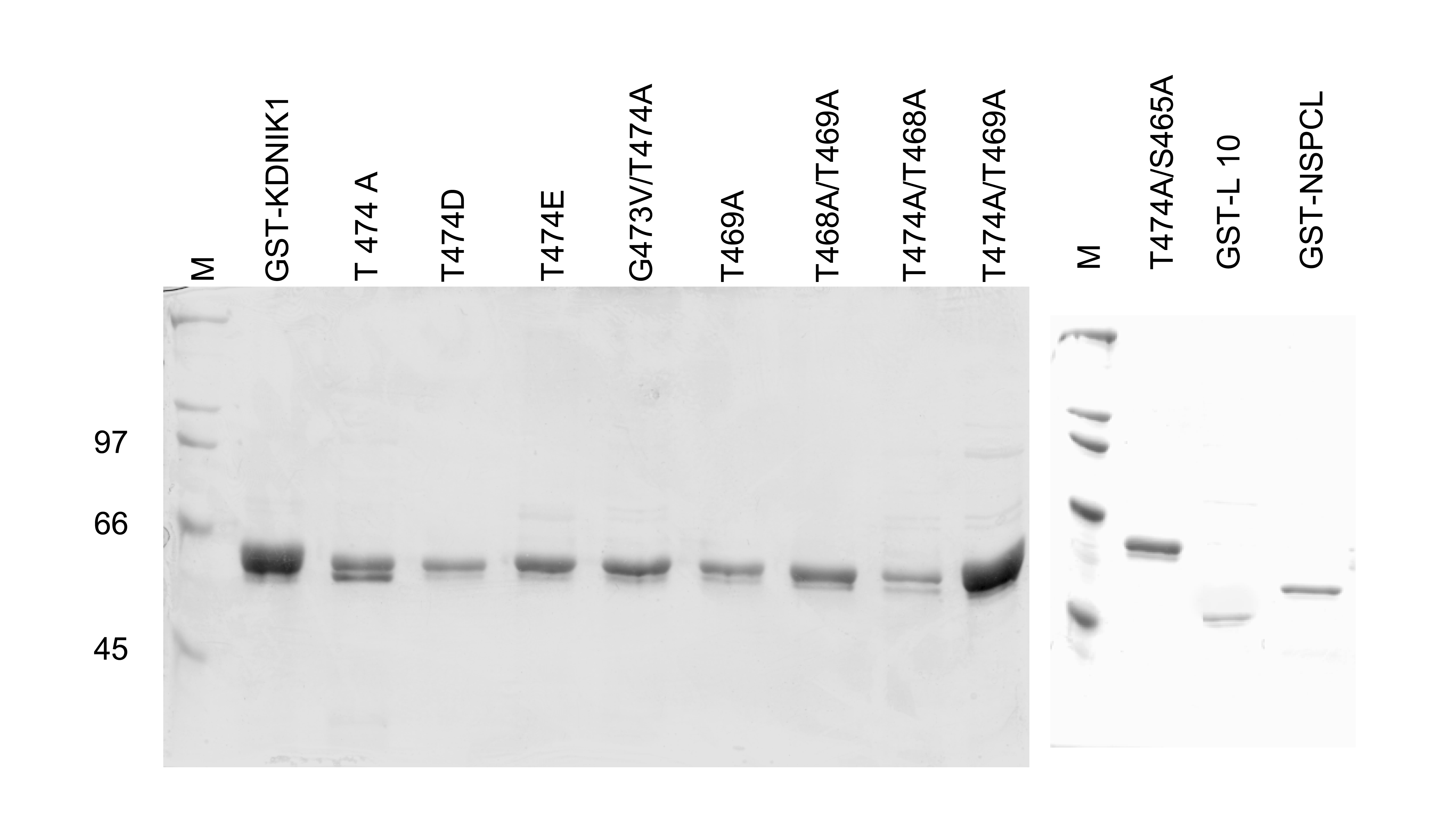

Supplement: Figure S1 — SDS-PAGE of E. coli- produced GST fusions. GST-fused to the C-terminal kinase domain of normal NIK1 (GST-KDNIK1) or to mutant NIK1s, as indicated, were produced in E. coli, affinity-purified, separated by SDS/PAGE and stained with coomassie brilliant blue. GST-L10 corresponds to a ribosomal protein L10 (rpL10) fused to GST and GST-NSP is a CaLCuV NSP fusion. Molecular mass markers (kDa) are shown on the left. (1.05 MB TIF) [file pone.0005781.s001.tif]

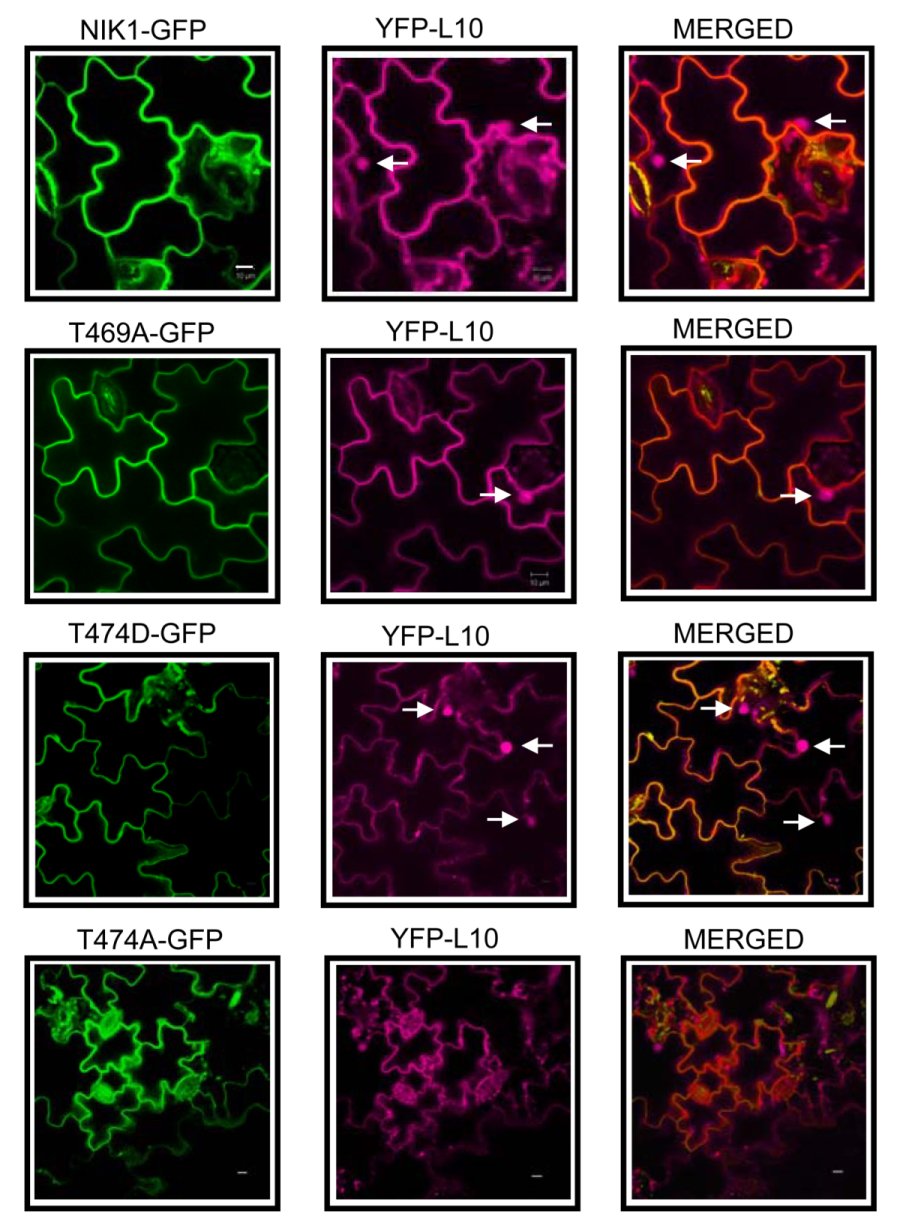

Supplement: Figure S2 — Effect of ectopic expression of NIK1 and A-loop mutants on nucleocytoplasmic shuttling of rpL10A. NIK1-GFP+YFP-L10, T469A-GFP+YFP-L10, T474D-GFP+YFP-L10 or T474A-GFP+YFP-L10 were co-expressed in tobacco leaf epidermal cells and the subcellular localization of the fluorescent fusion proteins was monitored by confocal microscopy. The frequency of co-transfected cells (merged field) with rpL10A localized within the nuclei was obtained. In each experiment, a total of 100 to 150 cells were observed. Full arrows indicate fluorescent nuclei. Scale bars are 10 µm (5.15 MB TIF) [file pone.0005781.s002.tif]
