# Supplementary material for: Conserved Threonine Residues within the A-Loop of the Receptor NIK Differentially Regulate the Kinase Function Required for Antiviral Signaling
Source: PLoS One. 2009 Jun 3;4(6):e5781. doi: 10.1371/journal.pone.0005781 (PMC2686266; doi:10.1371/journal.pone.0005781)
Supplement: Table S1 — Primers for mutagenesis within the kinase domain of NIK1 (0.04 MB DOC) [file pone.0005781.s003.doc]

Table S1- Primers for mutagenesis within the kinase domain of NIK1

| Mutants | Pair of primers |
| --- | --- |
| G473V/T474A | (+) 5’-TGTGACAACCGCGGTTAGAGTCGACGTGGGTCAC-3´ |
|  | (-) 5’-CTCTAACCGCGGTTGTCACATGAGAATCTT-3’ |
| T474E | (+) 5’-TGACAACCGCGGTTAGAGGCGAGGTGGGTCACAT-3’ |
|  | (-) 5’-GCCTCTAACCGCGGTTGTCACATGAGAATC-3’ |
| T474D | (+) 5’-TGACAACCGCGGTTAGAGGCGATGTGGGTCACAT-3’ |
|  | (-) 5’-GCCTCTAACCGCGGTTGTCACATGAGAATC-3’ |
| T474A | (+) 5’- TGACAACCGCGGTTAGAGGCGCGGTGGGTCACAT-3’ |
|  | (-) 5’-GCCTCTAACCGCGGTTGTCACATGAGAATC-3’ |
| T469A | (+) 5’- ATCAAGATTCTCATGTGACAGCCGCGGTTAGA-3’ |
|  | (-) 5’- TGTCACATGAGAATCTTGATGATCCAAGAG-3’ |
| T468A/T469A | (+) 5’- AAGATTCTCATGTGGCAGCCGCGGTTAGA-3’ |
|  | (-) 5’- CACATGAGAATCTTGATGATCCAAGAG-3’ |
| T474A/S465A | (+) 5’- ATCATCAAGATGCTCATGTGACAACC GCGGTTA-3’ |
|  | (-) 5’-ATCTTGATGATCCAAGAGTTTAGCTAAACC-3’ |
| T474A/T468A | (+) 5’-AAGATTCTCATGTGGCAACCGCGGTTAGA-3’ |
|  | (-) 5’- CACATGAGAATCTTGATGATCCAAGAG-3’ |
| T474A/T469A | (+) 5’-ATCAAGATTCTCATGTGACAGCCGCGGTTAGA-3’ |
|  | (-) 5’-TGTCACATGAGAATCTTGATGATCCAAGAG-3’ |
